# Editorial: Oxidative stress in ovarian follicles

**DOI:** 10.3389/fvets.2023.1126270

**Published:** 2023-02-09

**Authors:** Regiane R. Santos

**Affiliations:** Schothorst Feed Research, Lelystad, Netherlands

**Keywords:** oxidative stress, heat stress, *in vitro* culture, *in vivo* trial, plant extracts, melatonin

Several factors affect female reproduction, including factors related to oxidative stress caused by environmental heat stress (Ratchamak et al., Mutwedu et al.) or other stressors during the development of preantral (Silva et al.) and mature (Zhang et al.) ovarian follicles. Women are exposed to oxidative stress daily for the duration of their life, and this event will also interfere with their reproductive function. Therefore, studies on the impact of oxidative stress and antioxidant factors on female reproduction have been gaining interest. In addition to the necessary information on the effects of specific nutrients affecting animal reproduction, *in vitro* systems also need to be optimized by medium enrichment with antioxidants. This Research Topic, “*Oxidative stress in ovarian follicles*,” aimed to combine studies on different sources of oxidative stress and possible antioxidant strategies in laboratory and farm animals.

In the present Research Topic, four manuscripts cover the oxidative stress topic. The first two manuscripts evaluated heat stress on the ovaries of cattle (Ratchamak et al.) and the reproductive traits of rabbits (Mutwedu et al.), while the other studies are related to the *in vitro* analysis of ovarian preantral follicles (Silva et al.) and the subfertility of mice exposed to oxidative stress (Zhang et al.). Ratchamak et al. evaluated the effect of melatonin treatment on the ovarian response of dairy cows exposed to heat stress. For this, cycling Thai-Holstein dairy cows were superovulated under natural heat stress (ambient temperature of ~29.8°C). Superovulation was performed either combined with melatonin administration or without. These authors observed that melatonin increased ovarian response and the number of large follicles and corpora lutea. Furthermore, the transferable embryos from the melatonin-treated cows were greater than those from control cows. The antioxidant effect of melatonin was confirmed by a decrease in lipid peroxidation and increased activity of superoxide dismutase. When female rabbits were exposed to heat stress, Mutwedu et al. assessed the effect of a plant extract (*Moringa oleifera*) in decreasing the negative impact of this environmental challenge on the reproductive tract. Heat stress was artificially induced for 8 h per day while the rabbits were exposed to temperatures between 35–36°C. From the groups exposed to this high-heat stress, one of them received a gavage with an aqueous extract of *M. oleifera* for 80 days (200 mg once a day). The authors described a positive effect of this extract in the litter size and number and an increase in estradiol and follicle stimulating hormone (FSH) serum levels; however, increased ovarian follicular degeneration was also observed. Although the findings are promising for some parameters, dietary inclusion of *M. oleifera* could provide clearer information on the effects of this plant extract. Furthermore, a water extract is a mixture of several compounds that should also be evaluated individually.

Oxidative stress also occurs when ovarian follicles are cultured *in vitro*, either to be further used in reproduction programs or to be used as models for fertility studies. Silva et al. cultured goat preantral follicles enclosed in ovarian tissue in the presence of different antioxidants (eugenol, ascorbic acid, or anethole). Eugenol was able to protect the ovarian follicles from oxidative stress in the endoplasmic reticulum and decreased the production of reactive oxygen species. Assessment of the follicles included histological analysis and the expression of proteins and mRNA of markers related to oxidative stress and follicular function, as well as histone lysine methylation. These findings contribute to the improvement of the *in vitro* culture of immature oocytes enclosed in ovarian follicles, which could be used as reproductive reserves in fertilization programs. Finally, Zhang et al. demonstrated that ovarian oxidative stress is associated with subfertility in BALB/cMice. In brief, oxidative stress induces the depletion of the follicular population, and this effect can be counteracted with sodium selenite. This *in vivo* study revealed that follicular loss occurs by the disruption of the mitochondrial metabolism caused by oxidative stress, followed by the accumulation of reactive oxygen species and apoptosis.

The studies mentioned above provide substantial knowledge on the effects of oxidative stress and candidates to counteract its negative impact, which occurs when women are exposed to environmental stress (e.g., heat stress) or when female gametes are exposed to reactive oxygen species. The use of antioxidants in the culture medium can be directly applied and evaluated by further follicular development; however, nutritional intervention to counteract the impact of oxidative stress in female reproduction must deal with the inclusion of the compound of extract, chemical analysis when a blend is used, and palatability. The inclusion of a compound should neither interfere with the daily requirements for other nutrients nor exceed the level of the compound to avoid pro-oxidative activity. When using an extract, its chemical composition should be considered to avoid undesirable compounds or even contaminants/toxins. Finally, some studies use oral gavage to evaluate the effect of some antioxidants in the feed of animals; however, to follow practical conditions, dietary inclusion of antioxidants should reach a level that does not impair feed intake.

## Author contributions

The author confirms being the sole contributor of this work and has approved it for publication.

